# Supplementary material for: Pathogen avoidance and prey discrimination in ants
Source: R Soc Open Sci. 2020 Feb 19;7(2):191705. doi: 10.1098/rsos.191705 (PMC7062100; doi:10.1098/rsos.191705)
Supplement: supplementary tables [file rsos191705supp1.docx]

Supplementary material

TableS.1: Preliminary tests to check for consistency in ant responses between the first experimental series (i.e. 6 colonies) and the second experimental series (i.e. 4 colonies). Ant responses were compared for the Ctrl-LC and the Ctrl-Spo conditions. The n values represent the number of ants tested in each series for the considered experimental condition.


**Table S.2**: Sample size of ants used for each experimental conditions. Abbreviations: Ctrl: control; LC: prey covered with low concentration of conidia; HC: prey covered with high concentration of conidia; FKill: prey killed by the fungus; Dc: decaying prey; Spo: sporulating prey.

| Colonies | Ctrl-Ctrl | Ctrl-LC | Ctrl-HC | Ctrl-FKill | Ctrl-Spo | Dc-Spo |
| --- | --- | --- | --- | --- | --- | --- |
| A | 21 | 16 | 0 | 27 | 12 | 24 |
| B | 23 | 33 | 0 | 20 | 13 | 21 |
| C | 20 | 16 | 0 | 22 | 15 | 26 |
| D | 20 | 17 | 0 | 16 | 12 | 22 |
| E | 20 | 11 | 0 | 26 | 15 | 21 |
| F | 24 | 17 | 0 | 21 | 8 | 22 |
| G | 0 | 0 | 15 | 0 | 0 | 0 |
| H | 0 | 0 | 15 | 0 | 0 | 0 |
| I | 0 | 0 | 15 | 0 | 0 | 0 |
| J | 0 | 0 | 15 | 0 | 0 | 0 |
| Total number of tested ants | 128 | 110 | 60 | 132 | 75 | 136 |

**Table S.3** : Pairwise comparisons (Chi-squared test) of the percentage of ants retrieving a prey item for the different experimental conditions ( ns = non-significant; p>0.05).

|  | Ctrl - Ctrl | Ctrl - LC | Ctrl - HC | Ctrl - FKill | Ctrl - Spo | Dc - Spo |
| --- | --- | --- | --- | --- | --- | --- |
| Ctrl - Ctrl | - | - | - | - | - | - |
| Ctrl - LC | ns | - | - | - | - | - |
| Ctrl - HC | ns | ns | - | - | - | - |
| Ctrl - FKill | ns | ns | ns | - | - | - |
| Ctrl - Spo | <0.001 | <0.001 | ns | 0.005 | - | - |
| Dc - Spo | <0.001 | <0.001 | ns | <0.001 | ns | - |

**Table S.4** : Pairwise comparisons (Chi-squared test) of the percentage of ants contacting a single prey for the different experimental conditions ( ns = non-significant; p>0.05).

|  | Ctrl-Ctrl | Ctrl - LC | Ctrl - HC | Ctrl - FKill | Ctrl - Spo | Dc - Spo |
| --- | --- | --- | --- | --- | --- | --- |
| Ctrl - Ctrl | - | - | - | - | - | - |
| Ctrl - LC | ns | - | - | - | - | - |
| Ctrl - HC | ns | ns | - | - | - | - |
| Ctrl - FKill | ns | ns | ns | - | - | - |
| Ctrl - Spo | <0.001 | ns | ns | 0.02 | - | - |
| Dc - Spo | <0.001 | 0.008 | ns | <0.001 | ns | - |

**Table S.5** : Pairwise comparisons (Chi-squared test) of the percentage of ants retrieving prey item depending on its level of fungus infection, for ants that contacted a single prey. ( ns = non-significant; p>0.05).

|  | Control | Decaying prey | Low Conidia | High Conidia | Fungus-killed prey | Sporulating prey |
| --- | --- | --- | --- | --- | --- | --- |
| Control | - | - | - | - | - | - |
| Decaying | ns | - | - | - | - | - |
| Low Conidia | ns | ns | - | - | - | - |
| High Conidia | ns | ns | ns | - |  | - |
| Fungus-killed prey | ns | ns | ns | ns | - | - |
| Sporulating prey | <0.001 | 0.0025 | <0.001 | 0.016 | 0.0025 | - |

**Table S.6** : Pairwise comparisons (Chi-squared test) of percentage of no-choice between experimental conditions. P-adjustment= Bonferroni correction ( ns = non-significant; p>0.05).

|  | Ctrl - Ctrl | Ctrl - LC | Ctrl - HC | Ctrl - FKill | Ctrl - Spo | Dc - Spo |
| --- | --- | --- | --- | --- | --- | --- |
| Ctrl - Ctrl | - | - | - | - | - | - |
| Ctrl - LC | ns | - | - | - | - | - |
| Ctrl - HC | ns | ns | - | - | - | - |
| Ctrl - FKill | ns | ns | ns | - | - | - |
| Ctrl - Spo | 0.039 | ns | ns | ns | - | - |
| fK - Spo | 0.014 | 0.027 | ns | ns | ns | - |
